# Diagnosis of Periodontitis via Neutrophil Degranulation Signatures Identified by Integrated scRNA-Seq and Deep Learning

**DOI:** 10.3390/genes16091005

**Published:** 2025-08-26

**Authors:** Huijian Wu, Linqing Huang, Shuting Cai, Xiaoming Xiong, Yan He

**Affiliations:** 1 School of Advanced Manufacturing, Guangdong University of Technology, Jieyang 522000, China; wuhuijian@mails.gdut.edu.cn (H.W.); hlq@gdut.edu.cn (L.H.); 2 School of Integrated Circuits, Guangdong University of Technology, Guangzhou 510006, China; shutingcai@gdut.edu.cn (S.C.); xmxiong@gdut.edu.cn (X.X.); 3 School of Biomedical and Pharmaceutical Sciences, Guangdong University of Technology, Guangzhou 510006, China

**Keywords:** Periodontitis, Neutrophil degranulation, single-cell RNA-seq, machine learning, hdWGCNA, deep learning

## Abstract

**Background and objective**: Periodontitis, a chronic inflammatory disease driven by host immune dysregulation, leads to progressive destruction of periodontal tissues. This study employed an integrative approach combining single-cell transcriptomics, hierarchical weighted gene co-expression network analysis (hdWGCNA), and deep learning algorithms to identify key biomarkers associated with neutrophil degranulation in periodontitis, aiming to establish diagnostic models for early detection and precision interventions. **Methods**: We integrated single-cell RNA sequencing (scRNA-seq) data from human gingival tissues with bulk transcriptomic datasets. Pathogenic neutrophil subsets were characterized via pseudotime trajectory and cell–cell communication analyses. Hierarchical weighted gene co-expression network analysis (hdWGCNA) identified functional modules linked to degranulation. Machine learning and a convolutional neural network (CNN) model combining gene expression and immune cell profiles were developed for diagnosis. **Results**: scRNA-seq revealed a neutrophil subpopulation significantly increased infiltration in periodontitis, with cell–cell communication and pseudotime trajectory analyses demonstrating amplified inflammatory crosstalk. hdWGCNA identified the turquoise module enriched in PD-KEY-Neutrophils, containing hub genes linked to neutrophil degranulation and complement activation. Immune infiltration and non-negative matrix factorization linked high-degranulation neutrophil signatures to the periodontal immunity microenvironment. Machine learning demonstrated that the neutrophil degranulation-associated genes effectively distinguish diseased gingival tissue, suggesting their potential to predict periodontitis. Finally, integrating transcriptomic and immunological data, we developed a gene-immune CNN deep learning model accurately diagnosed periodontitis in diverse cohorts (AUC = 0.922). **Conclusions**: Our study identified a pathogenic neutrophil subpopulation driving periodontitis through degranulation and inflammation. The neutrophil degranulation genes serve as critical biomarkers, offering new insights for clinical diagnosis and treatment of periodontitis.

## 1. Introduction

Periodontitis is a chronic inflammatory oral disease initiated by dental plaque biofilms and host immune response [1]. Affecting over 45% of adults globally, it ranks as the sixth most prevalent human disease, severely compromising oral function and the quality of life [2]. It is noteworthy that severe periodontitis progression leads to irreversible alveolar bone loss, pathological tooth mobility, and eventually tooth loss, significantly impairing oral function and quality of life [3,4]. Moreover, emerging evidence underscores its systemic implications, with epidemiological studies linking periodontitis to the increased risks of systemic disease [5,6,7]. Therefore, early diagnosis and effective treatment of periodontitis are critical for maintaining overall health. Non-surgical periodontal therapy is the standard approach for managing periodontitis, with recent studies showing that adjunctive use of probiotics alongside NSPT can further improve clinical outcomes by modulating the host immune response and restoring microbial balance [8,9].

While bacteria plaque biofilms are recognized as a primary trigger of periodontitis, it is widely acknowledged that the host immune microenvironment dysregulation is central to periodontal pathogenesis [10,11,12]. Upon pathogenic bacterial invasion, the immune system activates to control infection, yet excessive responses might cause local or systemic chronic inflammation, leading to tissue destruction [13,14]. As the first responders to microbial challenges, neutrophils play a dual role: defending against pathogens while contributing to tissue damage [15,16]. Persistent neutrophil activation exacerbates tissue destruction in chronic inflammation. Growing evidence indicates that functional heterogeneity among neutrophils critically influences disease progression [16,17,18], yet specific pathogenic subpopulations and their mechanisms remain poorly characterized, leaving a gap in developing therapeutic modulation strategies [19].

Recent advances in single-cell transcriptomics have enabled high-resolution characterization of immune cell heterogeneity within periodontal lesions [20]. Hierarchical weighted gene co-expression network analysis (hdWGCNA) serves as a powerful approach for identifying gene modules characterized by correlated expression patterns, specially developed to handle the complexities of scRNA-seq data [21]. This study aims to integrate single-cell RNA sequencing and bulk RNA sequencing datasets to identify key neutrophil-associated signature genes in periodontitis through hdWGCNA and multiple machine learning algorithms, establishing diagnostic models for early detection and precision interventions.

## 2. Materials and Methods

### 2.1. Data Collection and Preprocessing

All gingival tissue datasets were obtained from the Gene Expression Omnibus (GEO) database (http://www.ncbi.nlm.nih.gov/geo/ (accessed on 25 August 2025)), including single-cell RNA sequencing (scRNA-seq) data GSE171213 and microarray data GSE16134 and GSE10334 (Supplementary Table S1) [22,23,24]. As previously described [25], for scRNA-seq data, cells expressing between 200 and 3000 genes with mitochondrial gene proportions below 20% were retained after quality control. Gene expression counts were normalized using a log-transformation, and the FindVariableFeatures function identified the top 2000 highly variable genes for scaling. Subsequent dimensionality reduction was performed via principal component analysis (PCA) and t-distributed stochastic neighbor embedding (tSNE) using FindNeighbors and FindCluster. Then, Cell types were annotated based on established marker genes [22]. For microarray datasets, “limma” package was utilized to eliminate batch effects [26].

### 2.2. Cell–Cell Communication and Pseudotime Analysis

To characterize neutrophil interactions within the periodontal immune microenviroment, we performed cell–cell communication analysis using the “CellChat” R package [27]. Ligand–receptor interactions were inferred from the curated CellChatDB database, and interaction strength and pathway importance scores were quantified using the computeCommunProb and netVisual_aggregate and netVisual_circle functions. Outgoing and incoming signaling interaction strengths in different cells was calculated using the netAnalysis_signalingRole with results depicted in heatmaps and scatter plots. Pathway-specific networks were further explored using circle plots and heatmaps to compare signaling activity between cell clusters. Pseudotime trajectory analysis was performed with the “Monocle2” package to infer developmental states of neutrophils in periodontitis and visualized this trajectory using the plot_cell_trajectory function [28].

### 2.3. hdWGCNA and Pathway Enrichment Analysis

Following package developer recommendations, the “hdWGCNA” R package was used to dissect transcriptional networks within the PD-KEY-Neutrophil subpopulation [21]. To optimize the scale-free topological model, the soft threshold (β) was set above 0.85 using TestSoftPowers function. The kME values were computed with the ModuleConnectivity function, where higher kME values indicated a stronger likelihood of a gene being a hub gene. The top hub genes within each module were identified using the GetHubGenes function, with the 50 genes exhibiting the highest kME values selected. Kyoto Encyclopedia of Genes and Genomes (KEGG) pathway analyses were subsequently conducted via the R package “clusterProfiler” and Metascape online website [29,30].

### 2.4. LASSO Regression and Machine Learning Algorithm

Microarray data (GSE16134 as training, GSE10334 as validation) were analyzed to develop a periodontitis diagnostic model based on neutrophil degranulation gene signatures from PD-KEY-Neutrophils. To effectively handle the high-dimensional transcriptomic data and prevent overfitting in our training cohort, we employed Least Absolute Shrinkage and Selection Operator (LASSO) regression to identify significant diagnostic genes, subsequently termed neutrophil degranulation-associated genes [31]. Seven supervised machine learning algorithms included k-nearest neighbor (KNN), logistic regression (LR), naïve Bayes (NB), random forest (RF), recursive partitioning and regression trees (RPART), support vector machine (SVM), and linear discriminant analysis (LDA), which were implemented using the “mlr3” R package [32]. Model robustness was assessed through ten iterations of 5-fold cross-validation on the training set, with performance metrics including area under the curve (AUC), sensitivity, specificity. To further validate the machine learning outcomes and investigate associations between the neutrophil degranulation-associated genes and immune cells, a convolutional neural network (CNN) was constructed [33]. The transcriptomic and immune profiles of each patient were encoded as an 18 × 10-unit 2D feature matrix (N_i,j_ = immune_i_/gene_j_) as input to the model. The CNN architecture initiated with a convolutional layer (32 filters, 3 × 3 kernel, ReLU activation) followed by feature extraction using 16 softplus-activated filters (2 × 2 kernel) coupled with max pooling (2 × 2 window). Flattened features were then fed into a fully-connected layer with 64 ReLU units, L_2_ regularization (λ = 0.02), and dropout regularization (rate = 0.5), culminating in a sigmoid output layer for periodontitis classification. The model training performed in Keras and TensorFlow frameworks [34,35].

### 2.5. Immune Infiltration Analysis and Non-Negative Matrix Factorization (NMF)

To characterize neutrophil degranulation-associated gene expression and immune microenvironment dynamics in periodontitis, differential expression of 18 neutrophil degranulation-related genes between periodontitis and healthy controls was assessed using the “limma” package with statistical significance set at adjusted *p* value < 0.05. Pairwise Pearson correlation coefficients among these genes were calculated to evaluate co-expression patterns, visualized through “ggplot2” and “heatmap” package. Neutrophil degranulation activity was quantified across samples via single-sample gene set enrichment analysis (ssGSEA) using the “GSVA” R package [36]. Additionally, The MCPcounter algorithm was applied to estimate the infiltration of immune cells, with correlation analysis between gene expression and immune cell proportions performed [36]. To stratify patients based on transcriptional heterogeneity, non-negative matrix factorization (NMF) was applied to gene expression data, with optimal cluster number determined by cophenetic coefficient maximization [37]. Subtype-specific differences in neutrophil degranulation scores and immune cell infiltration levels were statistically compared using the Wilcoxon rank-sum test.

### 2.6. Statistical Analysis

All analyses were conducted in R v4.2.2. Group comparisons were analyzed using the Wilcoxon rank-sum test for non-parametric data. Pearson correlation analysis was used to investigate the association between 18 neutrophil degranulation-related genes and immune cell infiltration. Statistical significance was defined as *p* < 0.05.

## 3. Results

### 3.1. Single-Cell RNA Sequencing Identified Expanded Neutrophil Subpopulations in Periodontitis

**Figure 1:**
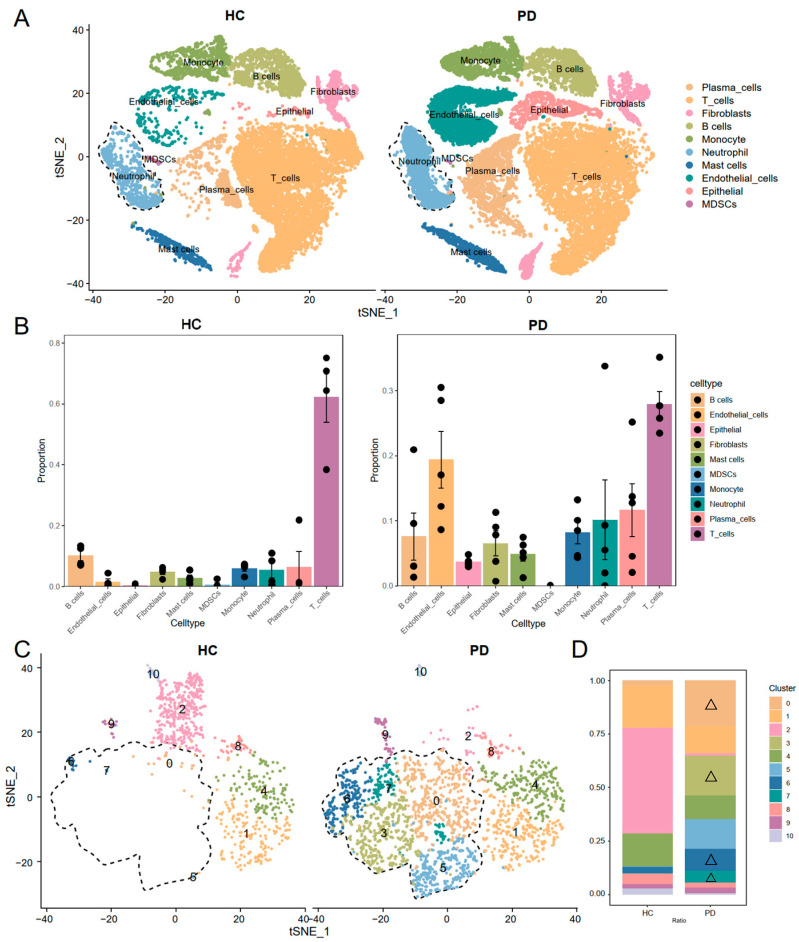
Cellular composition of gingival tissue in periodontitis and expanded neutrophil subpopulations was identified. (**A**) Annotation of 10 major cell types in gingival tissues based on canonical marker genes, including immune, stromal, and epithelial lineages. (**B**) Cell proportion analysis showing a significant expansion of immune cells, particularly neutrophils, in periodontitis tissues. (**C**) t-SNE plot illustrating the identification of subclusters of neutrophils. (**D**) Subclusters 0, 3, 5, 6, and 7 of neutrophils (marked with △ symbols) were enriched in periodontitis (labeled PD-key-neutrophils).

To explore the cellular composition and immune landscape of gingival tissue in periodontitis, we analyzed a representative scRNA-seq dataset (GSE171213), which encompasses gingival tissues from 4 healthy controls (HC) and 5 patients with severe chronic periodontitis (PD). Standard preprocessing steps, including stringent quality control, dimensionality reduction (t-SNE), and unsupervised clustering, were applied to integrate the dataset (Supplement Figure S1A–D). Following preprocessing, 36,054 high-quality cells were retained for downstream analysis and classified into 22 distinct cell clusters (Supplement Figure S1E). These clusters were manually annotated into 10 major cell types using established marker genes reported in prior studies, including immune, stromal, and epithelial lineages (Figure 1A, Supplement Figure S1F). Cell proportion analysis revealed a significant expansion of immune cell populations in periodontitis tissues, particularly neutrophils (Figure 1B). Given the well-documented yet incompletely understood role of neutrophils in periodontitis pathogenesis, we focused on neutrophils for sub-clustering and functional characterization. After clustering the neutrophils, subclustering of neutrophils identified 11 distinct subclusters (Figure 1C). Comparative analysis between healthy and diseased tissues revealed that subclusters 0, 3, 5, 6, and 7 were significantly enriched in periodontitis (Figure 1D). Consequently, these elevated subclusters were categorized as PD-associated neutrophils, while the remaining subpopulations were labeled as other-neutrophils for further investigation.

### 3.2. Cell–Cell Communication and Pseudotime Analysis of PD-Key-Neutrophil

**Figure 2:**
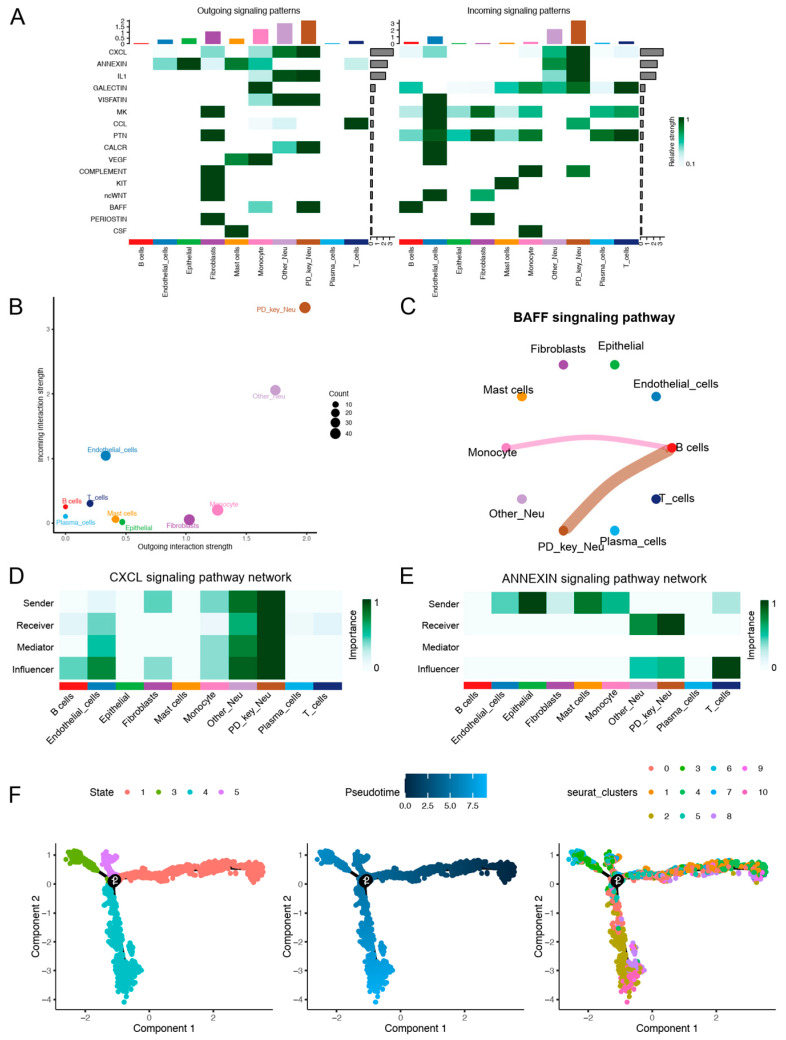
Cell–cell communication and pseudotime analysis of PD-key-neutrophil. (**A**) Heatmap showing the intensity of outgoing and incoming signals for all the cell types. (**B**) Scatter plot depicting the communication weights/strengths for various cell types, with PD-key-neutrophils showing significantly higher signaling strengths. (**C**) Circle plots showing BAFF signaling initiated by PD-KEY-Neutrophils and monocytes, absent in other neutrophil subpopulations. (**D**,**E**) Visualization of the roles of different cell types within the CXCL and ANNEXIN signaling pathways. (**F**) pseudotemporal trajectory analysis of the developmental stages of neutrophil subtypes. PD-KEY-Neutrophils (subclusters 0, 3, 5, 6, 7) localize to terminal branches, indicating their maturation state in advanced periodontitis.

Cell–cell interaction networks were constructed to identify the signaling pathways involved in the communication between PD-KEY-Neutrophils and other immune cells within the periodontitis inflammatory niche (Supplement Figure S2A). The analysis revealed all significant signaling pathways, highlighting key interactions (Figure 2A). Overall, Neutrophils exhibited the highest overall signaling activity, with PD-KEY-Neutrophils showing significantly higher signaling strengths than other neutrophil populations (Figure 2B). Notably, B cell-activating factor (BAFF) signaling was uniquely initiated by PD-KEY-Neutrophils and monocytes, absent in other neutrophils, implying their specialized capacity to amplify B cell responses in periodontitis (Figure 2C). The dominance of PD-KEY-Neutrophils was also evident in C-X-C motif chemokine ligand (CXCL) pathways, where they ranked highest as signal senders, receivers, and mediators (Figure 2D). Concurrently, enhanced ANNEXIN signaling toward PD-KEY-Neutrophils highlighted their role in modulating apoptotic processes within the inflammatory microenvironment (Figure 2E). Additionally, pseudotemporal trajectory analysis demonstrated the developmental stages of neutrophil subtypes during disease progression. The result suggested that PD-KEY-Neutrophils mainly localized to terminal branches (Figure 2F), suggesting their maturation was influenced by inflammatory cues in advanced periodontitis.

### 3.3. hdWGCNA Uncovered Turquoise Co-Expression Modules for PD-KEY-Neutrophil

**Figure 3:**
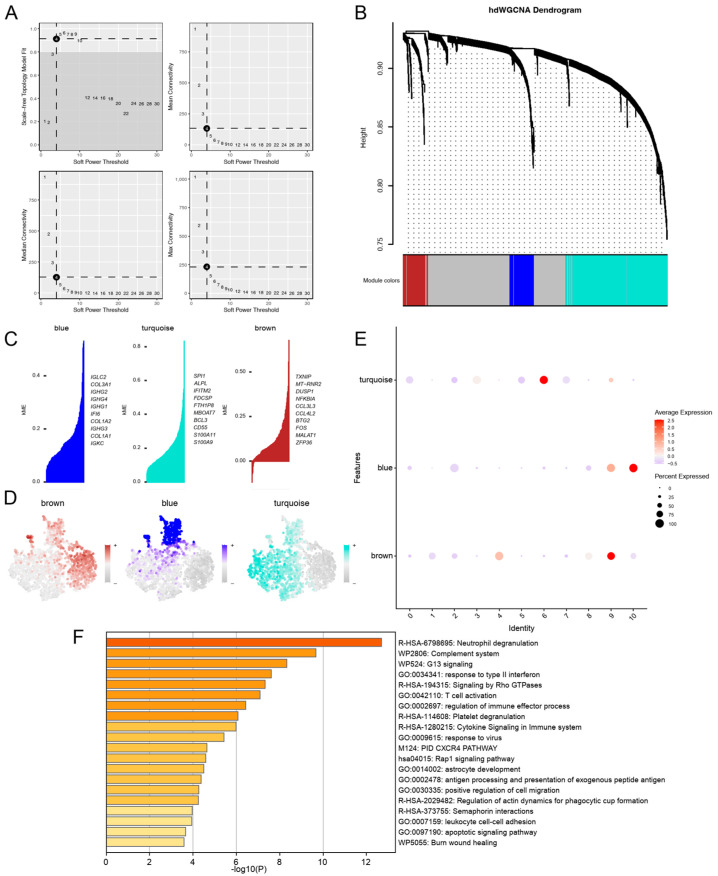
Identification of turquoise co-expression modules for PD-KEY-Neutrophils through hdWGCNA. (**A**) Soft power threshold of 4 was selected for hdWGCNA analysis. (**B**) Dendrogram from hdWGCNA identified three gene co-expression modules. (**C**) The top 10 genes for each co-expression module. (**D**) Feature Plots visualizing the expression patterns of these modules in neutrophils. (**E**) Dot plot showing selective enrichment of the turquoise module in PD-KEY-Neutrophils. (**F**) KEGG pathway enrichment analysis of the turquoise module genes. “Neutrophil Degranulation” (bolded) represents the most significantly upregulated pathway. “Complement Activation” (bolded) shows second-highest enrichment.

To uncover the characteristics and functions of neutrophils in periodontitis, we performed a hdWGCNA analysis. Soft power thresholding was applied to ensure the network’s scale-free topology, with an optimal threshold selected at 4 based on analysis of both mean connectivity and the standard error of the network (Figure 3A). We identified three gene co-expression modules through hierarchical clustering, represented by the colors blue, turquoise and brown (Figure 3B, Supplement Figure S2B). As shown in Figure 3C, hub genes were identified using kME method across co-expression modules, including IGLC2, COL3A1, IGHG2 in the blue module; SPI1, ALPL, IFITM2 in the turquoise module; and TXNIP, MT-RNR2, DUSP1 in the brown module. The expression patterns of these modules in neutrophils were further visualized through t-SNE plots (Figure 3D). Importantly, we found that the turquoise module exhibited selective enrichment in PD-KEY-Neutrophils (subclusters 0, 3, 5, 6, and 7, Figure 3E). Consequently, we suggested that the turquoise module might reflect characteristics of neutrophils in periodontitis. KEGG pathway enrichment analysis of the turquoise module revealed strong associations with neutrophil degranulation that complement system activation, alongside pathways central to tissue inflammatory and destruction (Figure 3F). Additionally, enrichment in CXCR4 signaling and Rho GTPase-mediated cytoskeletal remodeling underscored enhanced neutrophil migration and phagocytic activity [38,39]. Genes from the turquoise module were identified as possible periodontitis potential biomarkers.

### 3.4. Constructing a Machine Learning Diagnostic Model Leveraging Neutrophil Degranulation Gene Signatures

**Figure 4:**
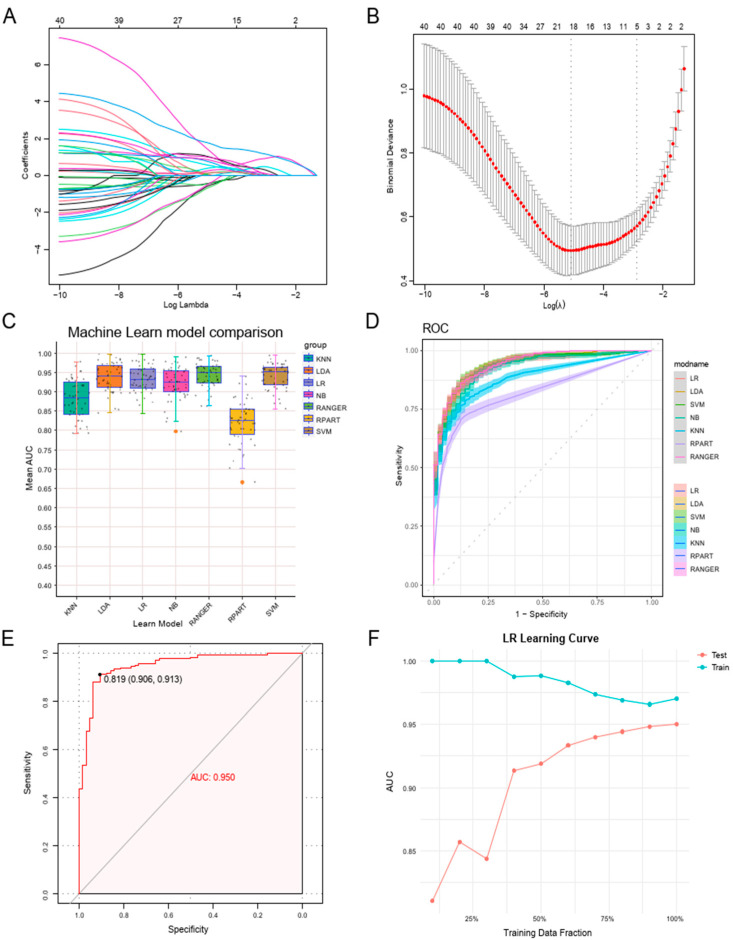
Machine learning diagnostic model was constructed using neutrophil degranulation gene signatures. (**A**,**B**) LASSO regression further selected 18 genes most strongly associated with neutrophil degranulation. Coefficient profile plots and penalty plot. (**C**) Diagnostic performance comparison of seven machine learning models. (**D**) Internal validation through 5-fold cross-validation for all models, with LR stood out with the best AUC, sensitivity, and specificity. (**E**) External validation of the LR algorithm achieving an AUC of 0.950. (**F**) Learning curve of the LR algorithm.

Considering the significant role of PD-KEY-Neutrophils in periodontitis, we constructed a diagnostic model using neutrophil degranulation gene signatures from PD-KEY-Neutrophils based on bulk RNA sequencing datasets. The GSE16134 dataset was used as the training set, while GSE10334 served as the validation. LASSO regression was applied to reduce dimensionality and select key genes, resulting in the identification of 18 genes most strongly associated with neutrophil degranulation in periodontitis (Figure 4A,B, Supplement Figure S2C). To evaluate their diagnostic performance, we integrated seven machine learning algorithms, including support vector machines (SVM), k-nearest neighbor (KNN), logistic regression (LR), naïve Bayes (NB), random forest (RF), recursive partitioning and regression trees (RPART), and linear discriminant analysis (LDA). We performed 10 repetitions of 5-fold cross-validation on the training set to assess the stability of each model. Most algorithms exhibited good stability across iterations, while KNN and RPART exhibited suboptimal performance, as indicated by lower AUC values and substantially higher false negative rates (Figure 4C and Supplementary Table S2). Further internal validation demonstrated that all models performed reasonably well, but LR stood out with the best AUC, sensitivity, and specificity (Figure 4D). For external validation, LR was selected as the final model and tested on the GSE10334 dataset, achieving an impressive AUC of 0.950, a sensitivity of 0.913, and a specificity of 0.906, confirming its strong predictive capability (Figure 4E,F).

### 3.5. Neutrophil Degranulation Genes Were Linked to Immune Profiles and Molecular Subtyping in Periodontitis

**Figure 5:**
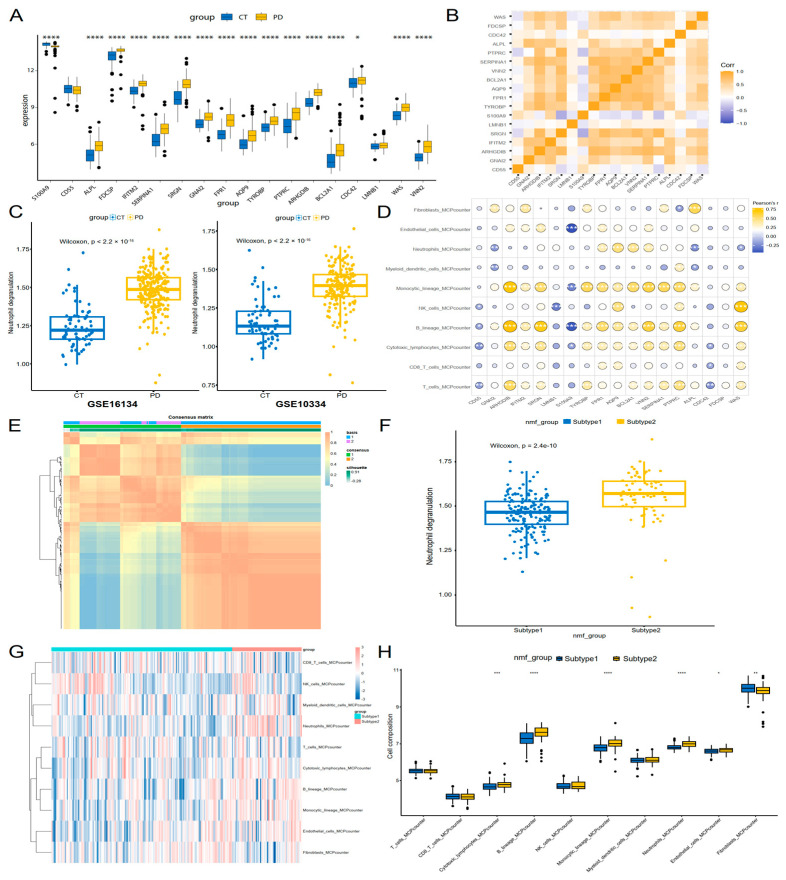
Immune infiltration and Subtyping analysis of periodontitis Patients. (**A**) Expression patterns of neutrophil degranulation-associated genes in periodontitis tissue. (**B**) Pearson correlation coefficient analysis among the neutrophil degranulation-associated genes. (**C**) Difference in neutrophil degranulation activity between periodontitis and control across two independent cohorts. (**D**) Heatmap of correlations between gene expression and immune cell infiltration (MCPcounter). Upregulated genes show strong positive correlations with monocytes, neutrophils, B cells, and cytotoxic lymphocytes. Conversely, downregulated S100A9 exhibits significant negative correlations (blue) with these populations. (**E**) Clustering of periodontitis patients into two subtypes. (**F**) Box plots showing differences in neutrophil degranulation activity between the two subtypes. (**G**,**H**) Heatmap and box plots showing the immune landscape between two subtypes. Wilcox.test or t.test was used to determine the significance of differences, with *p*.signif values of “*” < 0.05, “**” < 0.01, “***” < 0.001 and “****” < 0.0001.

The pathophysiology of periodontitis is significantly regulated by the immune microenvironment. To explore the interplay between neutrophil degranulation-associated genes and immune cell dynamics in periodontitis, we first analyzed the expression patterns of neutrophil degranulation-associated genes using periodontal tissue microarray data. Among 18 evaluated genes, most genes were significantly upregulated in periodontitis tissue compared to controls, while S100A9 was downregulated (Figure 5A, Supplement Figure S2D). Correlation analysis further revealed robust co-expression networks among upregulated genes, suggesting coordinated regulation of neutrophil-mediated inflammatory processes (Figure 5B). Functional assessment using ssGSEA highlighted elevated neutrophil degranulation activity in periodontitis tissue across two independent cohorts (Figure 5C). We utilized MCPcounter to comprehensively evaluate immune cell infiltration in gingival tissues of periodontitis patients. It was discovered that upregulated neutrophil degranulation-associated genes demonstrated strong positive correlations with infiltration levels of multiple immune cells, including monocytes, neutrophils, B cells and cytotoxic lymphocytes. In contrast, downregulated S100A9 exhibited significant negative correlations with the abundance of these immune populations (Figure 5D).

To uncover patient heterogeneity in periodontitis, the NMF algorithm was applied with neutrophil degranulation-associated genes, clustering periodontitis patients into two distinct subtypes (Figure 5E, Supplement Figure S2D). Compared to subtype 1, neutrophil degranulation activity in subtype 2 was significantly elevated (Figure 5F, Supplement Figure S2E). We further assessed the immune landscape between two subtypes by MCPcounter. The result revealed subtype 2 exhibited heightened myeloid cell (monocytic lineage cells and neutrophils), B cells and cytotoxic lymphocytes abundance, while fibroblasts showed decreased infiltration level (Figure 5G,H). These results suggested that subtype 2 may represent a more aggressive inflammatory phenotype, characterized by heightened neutrophil degranulation and immune activation, which could potentially influence disease progression and responsiveness to anti-inflammatory or immunomodulatory therapies.

### 3.6. Gene-Immune Convolutional Neural Network (CNN) for Diagnosis of Periodontitis

**Figure 6:**
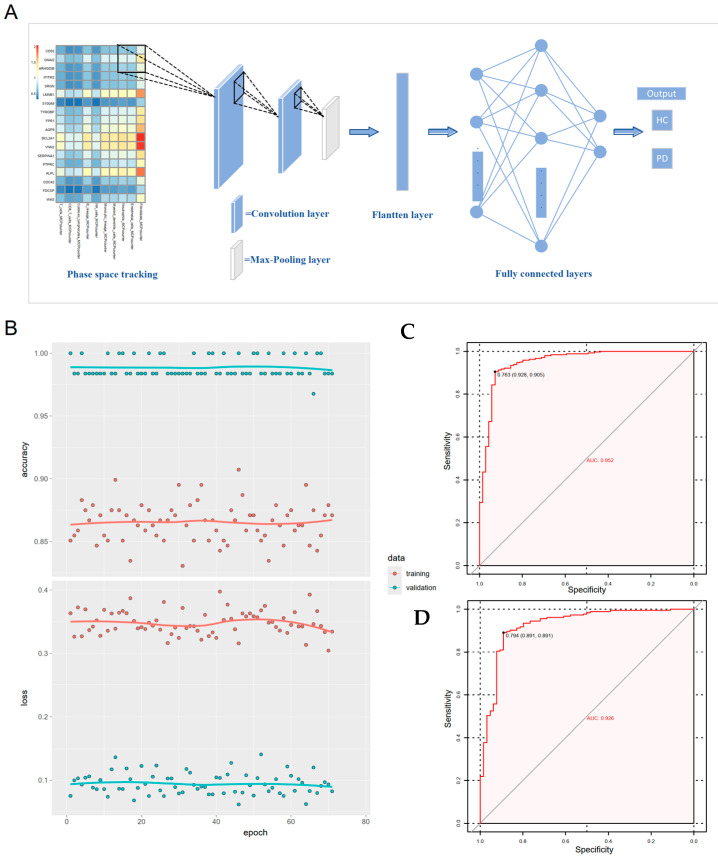
The gene-immune convolutional neural network (CNN) model for diagnosis of periodontitis. (**A**) schematic graphic illustrating the basic concept of gene-immune CNN model. (**B**) Training procedure of the gene-immune CNN model. (**C**,**D**) Performance of the gene-immune CNN model in both training and validation cohort.

To mitigate batch effects and integrate key neutrophil degranulation genes with immune microenvironment interactions in periodontitis, we constructed a gene-immune convolutional neural network (CNN) classifier. Patient-specific transcriptomic and immune cell profiles were encoded into a 2D heatmap (10 × 18 units), where each element represented the ratio between a specific gene’s expression level and the corresponding immune cell subset proportion (Figure 6A). Mirroring our machine learning framework, the GSE16134 cohort served as the training set, while GSE10334 was used for external validation. The CNN was trained for 100 epochs exhibited rapid convergence with stabilized loss and accuracy trajectories (Figure 6B). The model achieved exceptional diagnostic accuracy, with the training cohort yielding an AUC of 0.952, sensitivity of 0.905, and specificity of 0.928 (Figure 6C), and the validation cohort maintained high performance (AUC = 0.926, sensitivity = 0.891, specificity = 0.891, Figure 6D). These metrics paralleled the performance of traditional machine learning models while uniquely addressing batch effects through spatial feature learning, positioning the CNN as a versatile tool for periodontitis diagnosis with enhanced generalizability across heterogeneous cohorts.

## 4. Discussion

Periodontitis is characterized by a dysregulated host immune response that drives inflammatory tissue destruction [40]. Neutrophils, as the first line of defense against bacterial invasion, exacerbate tissue damage through excessive infiltration and dysregulated activation in this disease [41,42,43]. Clinical studies demonstrate that the severity of periodontitis is strongly correlated with peripheral and oral neutrophil count, highlighting neutrophils’ critical involvement in periodontitis progression [44,45]. Previous studies leveraging single-cell sequencing and flow cytometry in an independent patient cohort have confirmed elevated neutrophil infiltration in periodontitis tissues, consistent with our findings [46]. Specifically, we identified that several unique neutrophils subsets were distinct in periodontitis patients, termed as PD-key-neutrophils. Cell–cell interaction analysis revealed these subsets exhibited amplified signaling activity, particularly through BAFF and CXCL pathways. Notably, BAFF signaling promote B cell activation and maturation, whereas CXCL chemokines recruit monocytes and T cells, potentially sustaining chronic inflammation [47,48,49]. These findings highlight the particularly significant role of PD-key-neutrophils in the pathogenesis of periodontitis.

Neutrophils contribute to the development and progression of periodontitis through multiple mechanisms, shaped by interactions with dysbiotic oral bacteria. Pathogens such as *Porphyromonas gingivalis* promote neutrophil recruitment and activation via TLR2-PI3K signaling, inducing inflammatory cytokine production while suppressing phagosome maturation, thereby evading immune clearance and promoting bone resorption [50,51]. Concurrently, host neutrophils in periodontitis lesions exhibit hyperactivation, secreting chemokines (e.g., CXCL8) and cytokines (e.g., TNF-α and IL-1β), which amplify monocyte recruitment and chronic inflammation [52,53]. There cells also generate cytotoxic levels of reactive oxygen species (ROS) and elastase, which degrade collagen fibers and oxidize extracellular matrix proteins with massive neutrophil degranulation, directly contributing to connective tissue disintegration [54,55,56]. Furthermore, emerging evidence further underscores that excessive formation of neutrophil extracellular traps (NETs) is a crucial trigger of histopathological damage in periodontitis, which deepen periodontal pockets and accelerate bone resorption [57,58]. Recently, Single-cell RNA sequence analysis identified a NETs-related neutrophil subset in periodontitis gingival tissues and highlighted the pathogenic role of NETs in gingival inflammatory infiltration and alveolar bone resorption [59].

Neutrophils infiltrating periodontal tissues exhibit marked functional and transcriptional heterogeneity, with distinct activation states observed in health versus disease. Notably, the study also found that not all neutrophils moving through periodontal tissues were completely activated [19]. Moreover, a scoping review demonstrated that while the N1 or N2 neutrophil phenotypes remain unconfirmed in periodontitis, hyper-reactive proinflammatory neutrophils share key functional, including enhanced degranulation and NETosis [60]. In our study, the turquoise module was identified highly expressed in increased PD-key-neutrophils through hdWGCNA analysis, which were functionally linked to neutrophil degranulation and complement activation, reflecting their central role in periodontitis progression.

Neutrophil degranulation involves the release of pre-formed granules containing enzymes and mediators upon activation, with increased levels these markers have been found in periodontitis patients [61,62]. These granules contain destructive enzymes such as matrix metalloproteinases, gelatinase, myeloperoxidase, lactoferrin and elastase, which directly degrade collagen fibers in the periodontal ligament [63,64,65,66]. Furthermore, the destructive effects of degranulation are amplified by interactions with other neutrophil effector mechanisms. For instance, elastase primes reactive oxygen species ROS production, exacerbating oxidative damage to host tissues, while myeloperoxidase’s enzymatic processing of ROS is essential for neutrophil NET production, further propagating inflammation and epithelial barrier breakdown [67,68].

LASSO regression further identified 18 neutrophil degranulation-associated genes from transcriptomic datasets of periodontitis. These 18 key neutrophil degranulation genes were then applied to develop a periodontitis diagnostic model with robust accuracy cross both training and validation cohorts. Among these, *S100A9*, a gene encoding the pro-resolving calcium-binding protein calprotectin, regulates neutrophil chemotaxis, adhesion, and bactericidal activity, which plays a critical role in neutrophil-mediated immune responses [69]. Mendelian randomization studies demonstrated that elevated circulating neutrophil levels causally increase periodontitis risk, while S100A9 emerges as a protective factor against disease progression and therapeutic targets [70,71]. A cross-sectional study reported gingival crevicular fluid S100A9 levels were lower in established periodontitis patients compared to healthy controls, and S100A9 levels showed screening ability for periodontitis [72]. Meanwhile, four genes, including FPR1, AQP9, BCL2A1, and VNN2 showed significantly higher expression in periodontitis patients and were strongly correlated with neutrophil infiltration, emerging as central to diagnosis and understanding of periodontitis. FPR1, a bacterial peptide sensor, drives neutrophil chemotaxis toward inflamed sites, amplifying neutrophil recruitment in periodontitis [73]. Genetic predisposition due to FPR1 receptor polymorphisms has been identified as a critical determinant influencing interindividual variability in periodontitis and peri-implantitis susceptibility [74]. Recent evidence indicates that VNN2 expression positively correlates with neutrophil-related indicators and periodontal clinical parameters, suggesting its involvement in neutrophil-driven oxidative stress and epithelial barrier disruption [75]. However, the functional roles of AQP9 and BCL2A1 in periodontitis remain poorly understood, and further studies are needed to clarify their specific contributions to disease progression.

While our integrated bioinformatics approach provides novel insights into neutrophil degranulation signatures in periodontitis, several limitations warrant consideration. First, reliance on public transcriptomic datasets introduces inherent heterogeneity arising from variations in sampling protocols, sequencing platforms, and patient demographics across cohorts. Although our CNN model incorporated batch correction strategies, residual technical biases may confound the generalizability of the identified hub genes and neutrophil subpopulations. Notably, a key limitation is the exclusive use of computational models and public transcriptomics data without validation through protein-level assays or functional experiments; this constrains the biological robustness of key findings. Furthermore, the functional roles of identified genes such as AQP9 and BCL2A1, and their causal links to neutrophil-driven tissue destruction, could not be mechanistically interrogated in the absence of such functional assays. Finally, the exclusive focus on neutrophils does not account for potential crosstalk with other immune cell populations that may modulate the periodontal inflammatory microenvironment. Future research incorporating functional assays and in vitro experiments would provide crucial complementary mechanistic insights to strengthen the proposed diagnostic model.

## 5. Conclusions

In conclusion, this study identified a neutrophil subset with distinct degranulation signatures and critical biomarkers in periodontitis. By developing machine learning classifiers and deep neural networks, we established predictive models that delineate disease-associated neutrophil activation patterns. These findings provide a robust foundation for future research and the development of targeted therapeutic interventions to improve clinical outcomes.

## Data Availability

The data employed to support the findings of this study are freely available on the Gene Expression Omnibus (GEO) database, which houses extensive genomic sequencing and gene expression microarray data (https://www.ncbi.nlm.nih.gov/geo/ (accessed on 25 August 2025)).

## Supplementary Materials

The following supporting information can be downloaded at: https://www.mdpi.com/article/10.3390/genes16091005/s1, Table S1: Summary description table for datasets GSE171213, GSE16134 and GSE10334; Table S2: Performances of seven machine learning methods; Figure S1: scRNA-seq data preprocessing and cell type annotation in periodontal tissue. A: The nFeature_RNA, nCount_RNA, and percent.mt of each cell after quality control. B: Distribution of gene counts (nFeature_RNA) per sample. C: The scatter plot showing the link between nCount_RNA and nFeature_RNA. Pearson correlation coefficients are shown by the numbers above the graph. D: Elbow plot for selecting principal components (PCs) based on variance explained. E: t-SNE visualization of 22 distinct cell clusters. F: Dot plot of key marker gene expression across clusters; Figure S2: A: Network of cell communication, number of interactions (left); Strength of interactions (right). B: Correlation analysis among the three different gene modules. C: Normalized dispersion plot of genes, highlighting highly variable genes. D: Non-negative matrix factorization clustering rank survey. E: Differential expression of neutrophil degranulation-associated genes across subtypes.

## Abbreviations

The following abbreviations are used in this manuscript:

hdWGCNA	Hierarchical weighted gene co-expression network analysis
CNN	Convolutional neural network
scRNA-seq	Single-cell RNA sequencing
PD	Periodontitis
HC	Healthy controls
SVM	Support vector machines
KNN	K-nearest neighbor
NB	Naïve Bayes
RF	Random forest
RPART	Recursive partitioning and regression trees
LDA	Linear discriminant analysis
GEO	Gene Expression Omnibus
KEGG	Kyoto Encyclopedia of Genes and Genomes
