# Supplementary material for: Diagnosis of Periodontitis via Neutrophil Degranulation Signatures Identified by Integrated scRNA-Seq and Deep Learning
Source: Genes (Basel). 2025 Aug 26;16(9):1005. doi: 10.3390/genes16091005 (PMC12469482; doi:10.3390/genes16091005)
Supplement: Supplementary file 1 [file genes-16-01005-s001.zip › Supplementary materials/SF1.pdf]

Supplementary table 1. Summary description table for datasets GSE171213, GSE16134 and GSE10334.

	Platform	Species	Tissue	Samples in PD	Samples in CT	Reference
GSE171213	GPL24676	Homo sapiens	gingival tissue	5	4	PMID: 35154475
GSE16134	GPL570	Homo sapiens	gingival tissue	241	69	PMID: 24646639
GSE10334	GPL570	Homo sapiens	gingival tissue	183	64	PMID: 18980520

Supplementary table 2. Performances of 7machine learning methods.

Method	AUC	Sensitivity	Specificity	FNR	FPR
LR	0.931276	0.726044	0.951003	0.273956	0.048997
LDA	0.937094	0.669231	0.959728	0.330769	0.040272
SVM	0.939815	0.656044	0.960536	0.343956	0.039464
NB	0.920535	0.719121	0.924447	0.280879	0.075553
KNN	0.881241	0.492857	0.97466	0.507143	0.02534
RPART	0.820039	0.60033	0.920298	0.39967	0.079702
RANGER	0.941985	0.652747	0.967177	0.347253	0.032823
