# Supplementary figures and images for: Diagnosis of Periodontitis via Neutrophil Degranulation Signatures Identified by Integrated scRNA-Seq and Deep Learning
Source: Genes (Basel). 2025 Aug 26;16(9):1005. doi: 10.3390/genes16091005 (PMC12469482; doi:10.3390/genes16091005)

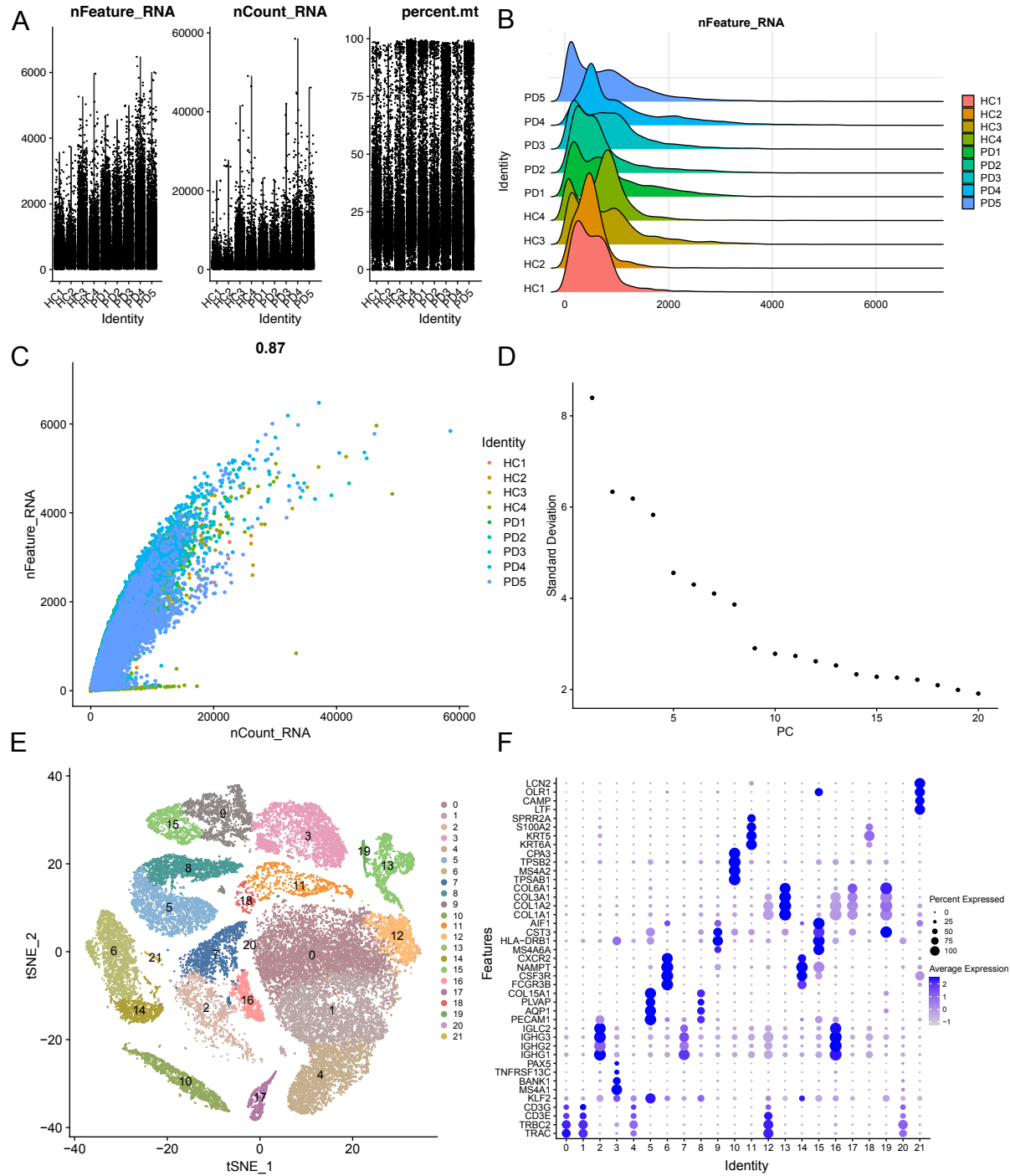

Supplement: Supplementary file 1 [file genes-16-01005-s001.zip › Supplementary materials/SF2.pdf]

A

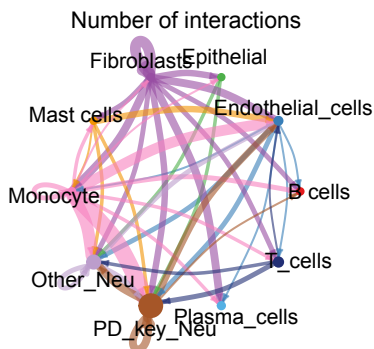

Interaction weights/strength

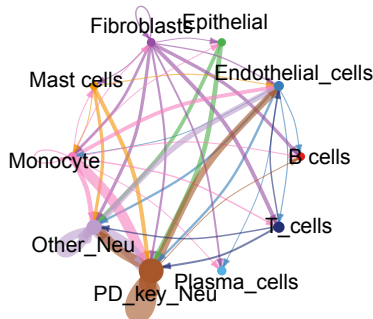

B

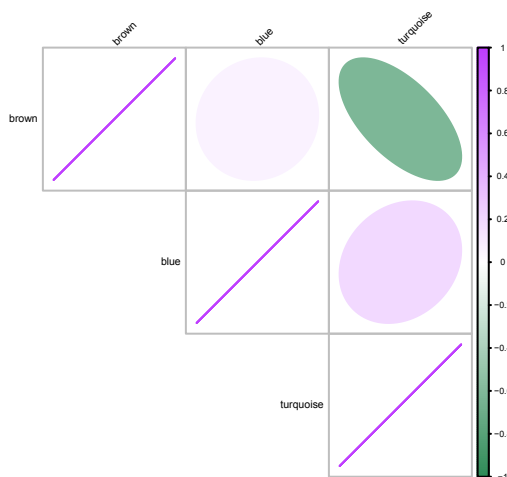

C

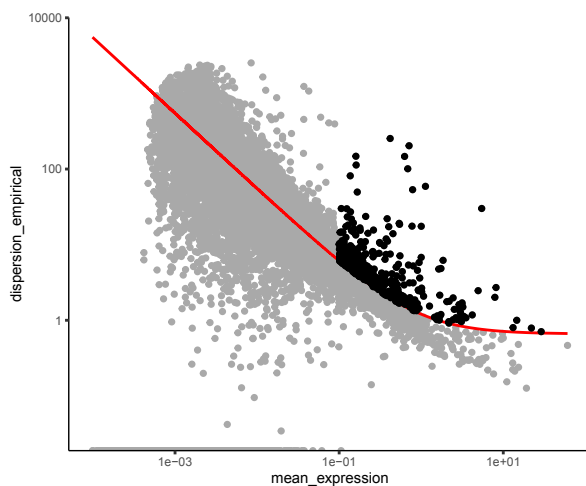

D

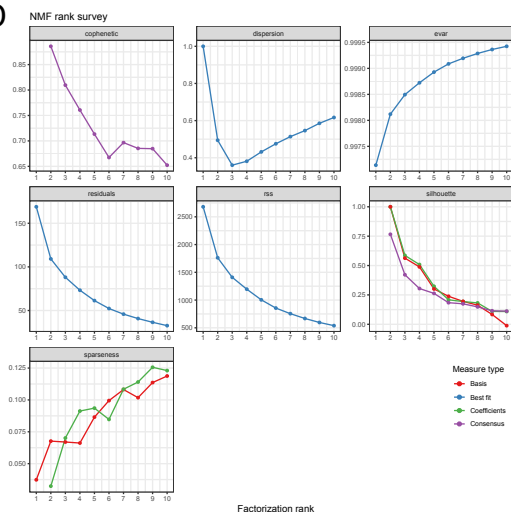

E

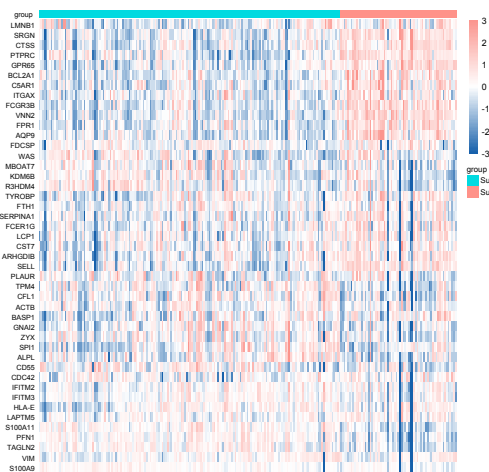

Supplement: Supplementary file 1 [file genes-16-01005-s001.zip › Supplementary materials/SF3.pdf]
